# Supplementary material for: Survival of Breast Cancer by Stage, Grade and Molecular Groups in Mallorca, Spain
Source: J Clin Med. 2022 Sep 27;11(19):5708. doi: 10.3390/jcm11195708 (PMC9571737; doi:10.3390/jcm11195708)
Supplement: Supplementary file 1 [file jcm-11-05708-s001.zip › jcm-1783737-supplementary.pdf]

Journal of
Clinical Medicine

MDPI

Table S1. Description of breast cancer cases by age group (N = 2869).

Variable	Categories	Number	%
Age	15-49	853	29.7
	50-69	1228	42.8
	70 or more	786	27.4
	Missing	2	0.1

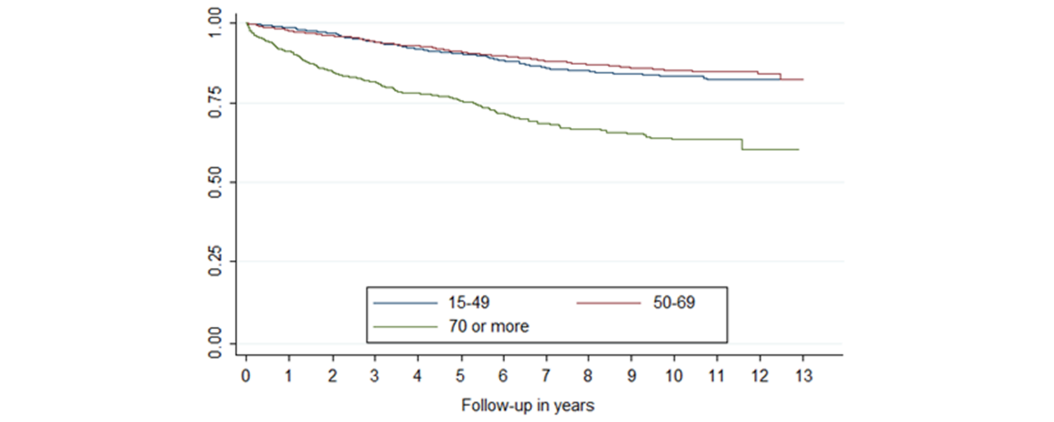

Figure S1. Survival curves of breast cancer by age (log-rank test: $\chi2(2)$=152.31, p<0.001).

Table S2. Competing-risks regression model of breast cancer before (Model 1) and after (Model 2) multiple imputation (MI) (m = 5).

	Model 1 (Original data set) N = 1924				Model 2 (Imputed data set) N = 2787			
**Variables**	**Subhazard ratio**	**St. Err.**	***p***	**CI 95%**	**Subhazard ratio**	**St. Err.**	***p***	**CI 95%**
**Age (ref. 15-49)**								
50-69	1.03	0.14	0.846	0.79, 1.34	0.94	0.11	0.627	0.74, 1.19
70 or more	1.99	0.28	<0.001	1.51, 2.64	1.96	0.25	<0.001	1.52, 2.51
**Sublocation (ref. Extern lower quadrant)**								
Nipple / Central region	1.12	0.32	0.679	0.65, 1.95	1.02	0.26	0.946	0.61, 1.69
Intern upper quadrant	1.96	0.56	0.019	1.12, 3.44	1.23	0.31	0.409	0.75, 2.03
Intern lower quadrant	1.04	0.38	0.933	0.50, 2.12	1.04	0.45	0.924	0.41, 2.66
Extern upper quadrant	1.36	0.33	0.201	0.85, 2.18	1.11	0.24	0.630	0.73, 1.68
Axillary	0.41	0.24	0.129	0.13, 1.29	0.57	0.57	0.590	0.05, 6.06
More than one location	1.57	0.37	0.056	0.99, 2.49	1.20	0.26	0.389	0.79, 1.84
**Stage (ref. IA)**								
IIA	2.31	0.57	0.001	1.42, 3.76	1.85	0.46	0.025	1.09, 3.13
IIB	3.28	0.82	<0.001	2.00, 5.36	2.48	0.62	0.001	1.48, 4.17
IIIA	8.26	1.96	<0.001	5.18, 13.14	5.38	1.42	<0.001	3.06, 9.47
IIIB	9.51	2.75	<0.001	5.40, 16.75	6.39	2.04	<0.001	3.26, 12.54

IIIC	12.06	3.46	<0.001	6.87, 21.16	7.68	2.50	<0.001	3.82, 15.43
IV	42.93	10.22	<0.001	26.92, 68.46	21.36	5.10	<0.001	12.96, 35.18
**Laterality (ref. Left)**								
Right	1.22	0.15	0.100	0.96, 1.55	1.10	0.12	0.373	0.89, 1.36
Bilateral	1.85	0.38	0.003	1.23, 2.78	1.41	0.41	0.256	0.75, 2.65
**Molecular classification (ref. Luminal with Ki67 unknown)**								
Luminal A	0.41	0.10	<0.001	0.26, 0.65	0.63	0.22	0.229	0.27, 1.44
Luminal B	1.00	0.15	0.984	0.75, 1.33	1.02	0.19	0.914	0.69, 1.52
Her-2 enriched	1.46	0.33	0.093	0.94, 2.28	1.30	0.29	0.247	0.82, 2.06
Triple negative	2.53	0.40	<0.001	1.85, 3.46	1.82	0.29	<0.001	1.32, 2.51
